# HADHA alleviates hepatic steatosis and oxidative stress in NAFLD via inactivation of the MKK3/MAPK pathway

**DOI:** 10.1007/s11033-022-07965-2

**Published:** 2022-11-14

**Authors:** Jiexia Ding, Lili Wu, Guoxian Zhu, Jing Zhu, Pingping Luo, Youming Li

**Affiliations:** 1 Department of Infectious Diseases, Affiliated Hangzhou First People’s Hospital, Zhejiang University School of Medicine, No. 261 Huansha Road, 310003 Hangzhou, Zhejiang Province China; 2 Department of Oncology, Ruian City People’s Hospital, 325200 Rui’an, China; 3 Department of Gastroenterology, Zhejiang University School of Medicine First Affiliated Hospital, 310003 Hangzhou, China

**Keywords:** HADHA, Nonalcoholic fatty liver disease, Lipid metabolism, Oxidative stress, MKK3/MAPK

## Abstract

**Background:**

Nonalcoholic fatty liver disease (NAFLD) is a liver metabolic syndrome and still lacks effective treatments because the molecular mechanism underlying the development of NAFLD is not completely understood. We investigated the role of Hydroxyl CoA dehydrogenase alpha subunit (HADHA) in the pathogenesis of NAFLD.

**Methods:**

HADHA expression was detected both in NAFLD cell and mice, and knockdown of HADHA in free fatty acids (FFA)-treated L02 or overexpression of HADHA in high fat diet (HFD)-fed mice was used to detected the influence of HADHA on hepatic steatosis, mitochondrial dysfunction, and oxidative stress by regulating of MKK3/MAPK signaling.

**Results:**

Our data revealed that HADHA expression was decreased in FFA-treated L02 cells and in HFD-fed mice. Knockdown of HADHA markedly aggravated hepatic steatosis, inflammation and oxidative stress in FFA-treated L02 cells, which was associated with the activation of MKK3/MAPK signalling pathways. Moreover, oxidative stress and liver lesions were improved in NAFLD mice by upregulation of HADHA. Importantly, we demonstrated that overexpression of HADHA inhibited the expression of p-MAPK in NAFLD mice, reducing lipid accumulation and steatosis.

**Conclusion:**

HADHA may function as a protective factor in the progression of NAFLD by alleviating abnormal metabolism and oxidative stress by suppressing MKK3/MAPK signalling pathway activation, providing a new target for the treatment of NAFLD.

## Introduction

Nonalcoholic fatty liver disease (NAFLD) is initialized by abnormal lipid accumulation in hepatocytes in the absence of alcohol intake 1. According to different developmental processes and pathological changes, NAFLD can be divided into three stages. The earliest stage is simple fatty liver. At this stage, there is excessive fat deposition in liver cells, but it has not yet caused liver cell damage. The second stage is non-alcoholic steatohepatitis (NASH), with liver cell damage and inflammatory cell infiltration. In the third stage, intrahepatic fibrosis or even cirrhosis occurs. Therefore, NAFLD is also the pathophysiological basis of many diseases (steatohepatitis, liver fibrosis, cirrhosis and hepatocellular carcinoma) and threatens human health 2, 3. Aside from lifestyle modification and weight loss, there is no effective treatment for NAFLD 4, 5. The formation of large lipid droplets in hepatocytes is caused by excessive inflammation, endoplasmic reticulum (ER) stress and oxidative stress 6. NAFLD mouse livers show oxidative damage, excessive inflammation and liver damage 7. ER stress triggers the unfolded protein response (UPR), resulting in inflammation and inflammasome activation of hepatocytes 8.

The mitogen-activated protein kinase (MAPK) signalling pathway is fundamental in inflammation and oxidative stress because it regulates nuclear factor E2-related factor 2 (Nrf2) and nuclear factor kappa B (NF-κB), which are involved in liver and metabolic diseases 9, 10. Serum vitamin D deficiency (VDD) patients show upregulation of p-MAPK expression in the serum 11. Activation of MAPK signalling in high fat diet (HFD)-fed mice contributes to lipid accumulation and inflammatory and reactive oxygen species (ROS) production 12. The R4/B subfamily regulators of G protein signalling protein 5 (RGS5) inhibit the activation of JNK/p38 pathways, which is a promising target for NAFLD 13, 14. The inactivation of NF-κB and MAPK cascades reverses hepatic steatosis, inflammation, and abnormal lipid metabolism, representing a potential strategy for NAFLD treatment 15. Lipid accumulation and inflammatory responses are triggered by MAPK signalling to exacerbate NAFLD progression 16. Activation of MAPK modulates lipid metabolism-related gene expression to participate in NAFLD progression 17. Insulin resistance is induced by activation of MAPK to promote NAFLD development 18. Lipid accumulation is induced by an ERK1/2-dependent pathway to promote NAFLD development 19.

Mitogen-activated protein kinase kinase 3 (MKK3) activates MAPK to regulate inflammation 20, oxidative stress 21 and ER stress 22. Phosphorylation of MKK3 (p-MKK3) is increased in the NAFLD mouse liver, and melatonin alleviates NAFLD phenotypes such as body weight gain, hepatic lipid accumulation, and fibrosis by inhibiting p-MKK3 expression 23. MKK3 upregulates TNF-α production to exacerbate inflammation, which results in liver damage 24. However, the regulatory mechanism of the MKK3 pathway in NAFLD progression is still unknown.

Hydroxyl CoA dehydrogenase alpha subunit (HADHA) regulates fatty acid beta-oxidation 25, lipid programming 26 and mitochondrial function 27, which are involved in inflammatory and oxidative stress response. Recent research findings show that acetylation of the mitochondrial β-oxidation enzyme HADHA modulates hepatic fatty acid oxidation activity, and HADHA may be a key regulator during the pathogenesis of fatty liver disease 28. The diabetic myocardium decreases the activity of HADHA to promote lipid droplet accumulation and elevate ER stress 29. The decreased expression of HADHA in NASH liver was verified by western blotting, which was used as a complementary technique to confirm the proteomic results 30. Nevertheless, the beneficial role of HADHA in NAFLD remains unclear.

Here, our study showed that HADHA expression was decreased in both free fatty acid (FFA)-treated L02 cells and NAFLD mouse liver tissues. In addition, HADHA overexpression in NAFLD mice inhibited the progression of hepatic steatosis. Mechanistic studies demonstrated that HADHA inactivated the MAPK pathway. Upregulation of HADHA alleviated lipid accumulation and oxidative stress in NAFLAD mice. Knocking down HADHA aggravated the damage caused by NAFLD. In summary, our findings reveal a previously unappreciated role of HADHA in NAFLD development, which may be a potential therapeutic target for NAFLD.

## Materials and methods

### Cell line culture and treatment

L02 normal human liver cells were purchased from the ATCC and cultured in RPMI 1640 medium (Thermo Scientific™, # 88,365) with 10% foetal bovine serum (Gibco™, #30,044,333) and 1% penicillin/streptomycin in a humidified atmosphere with 5% CO2 at 37 °C. We used 1 mM FFA (FFA; oleate acid and palmitate acid (2:1)) to treat L02 cells to establish a NAFLD cell model. L02 cells were cultured in RPMI 1640 medium containing FBS until the cells reached 80% confluence and then treated with 1 mM FFA for 24 h (24 h). The cells were transfected with 25 nM siHADHA or siControl for 24 h. The siRNA sequence for HADHA was 5`-TGGTGACAAGATTTGTGAA-3`. The siRNA sequence for the control was 5`-TTCTCCGAACGTGTCACGT-3`.

### Animals and treatment

Adenovirus (Adv) vectors were used to drive the expression of GFP (Adv-NC) or HADHA (Adv-HADHA) in mouse livers. Eight-week-old male C57BL/6 mice were randomly divided into 4 groups: the control group was fed a standard chow diet; the NAFLD model group was fed a HFD for 8 weeks (8 w); the HFD + Adv-NC group was fed a HFD for 6 weeks and then administered 2 × 10^9^ ifu Adv-NC by tail vein injection; and the HFD + Adv-HADHA group was fed a HFD for 6 weeks and then administered 2 × 10^9^ ifu Adv-HADHA by tail vein injection. Two weeks after virus injection, the mice were sacrificed using isoflurane. All animal studies were approved by the Animal Care and Use Committee of Zhejiang University in accordance with the Chinese guidelines for the care and use of laboratory animals.

### Western blot assay

RIPA lysis buffer (Thermo Scientific™, 89,900) containing PMSF (Thermo Scientific™, #36,978) was used to lyse L02 cells. The BCA method (Thermo Scientific™, #23,225) was used to measure the concentration of the protein lysate. After mixing 60 µg of cell lysate with 5× sample buffer, SDS–PAGE was used to separate the proteins, and the separated proteins were transferred to a PVDF membrane (Bio-Rad, #162–0177). After blocking with 4% milk containing 0.1% Tween, the following antibodies were added, and the membrane was incubated overnight at 4 °C: phosphor-MKK3 (1:2000, Cell Signaling Technology, #9231), MKK3 (1:2000, Cell Signaling Technology, #5674), HADHA (1:2000, Abcam, ab203114), phosphor-MAPK (1:1000, Cell Signaling Technology, #4370), MAPK (1:2000, Cell Signaling Technology, #9102), and GAPDH (1:1500, Abcam, ab9485). After washing the membrane 3 times with PBS solution containing 0.1% Tween, 4% milk containing 0.1% Tween and an HRP secondary antibody (1:4000; Abcam, ab205718)) were added, and the membrane was incubated for 2 h at room temperature. The membrane was removed, and ECL reagent (Bio-Rad, 170–5060) was added to the membrane. The membrane was placed into a Micro-Chemi 4.2 imaging system (Bio-Rad). ImageJ software was used to perform optical density analysis.

### qRT–PCR assays

TRIzol reagent (Invitrogen, #12,183,555) was used to extract total RNA. cDNA synthesis was performed using a high-capacity cDNA reverse transcription kit (Applied Biosystems, #4,368,813). The qRT–PCR experiment was performed using a StepOnePlus Real-Time PCR system with the SYBR Green experimental method (Applied Biosystems). The relative expression of genes was calculated by the 2^−ΔΔCt^ method. The qRT–PCR primer sequences and related primer sequences are listed in Table 1.

**Table 1:** qRT–PCR primer sequences and related primer sequences

Gene	Forward Primer (5`-3`)	Reverse Primer (5`-3`)
Mus-HADHA	TGCATTTGCCGCAGCTTTAC	GTTGGCCCAGATTTCGTTCA
Human-HADHA	TCAAGCAGGGGAAGGTCA	CTGGAGGATTCGGATGACTT
Human-PPARα	GCGAGCTCGCCTCCCTGTTGTTTCTA	GCGTCGACGGTGGCATCAGTCTTCAT
Human-CPT2	CATACAAGCTACATTTCGGGACC	AGCCCGGAGTGTCTTCAGAA
Human-GAPDH	TCAAGAAGGTGGTGAAGCAGG	TCAAAGGTGGAGGAGTGGGT
Human-EHHADH	AAACTCAGACCCGGTTGAAGA	TTGCAGAGTCTACGGGATTCT
Human-ECHS1	TGTCCTGTTGAGACACTGGTG	ACAAACGCGGTCATCCCTTC
Human-HADHB	CTGTCCAGACCAAAACGAAGAA	CGATGCAACAAACCCGTAAGC
Human-HADH	CACACAGTAGTGTTGGTAGACC	TGCCACTTTCCTAAGGCTTTC
Human-ACOX1	TGTCCTATTTGAACGACCTGCCCA	AGGTTCCAAGCTACCTCCTTGCTT
Mus-GAPDH	AGGCCGGTGCTGAGTATGTC	TGCCTGCTTCACCACCTTCT

### Oil red O staining in L02 cells and liver tissues

Cells on slides were fixed with 4% paraformaldehyde and Oil red O staining solution (Nanjing Jiancheng Bioengineering Institute (NJBI), D027) for 15 min, washed in 60% isopropanol and counterstained with haematoxylin after rinsing in distilled water.

### Detection of mitochondrial membrane potential (MMP) in L02 cells and liver tissues

Mitochondrial depolarization was analysed with an MMP assay kit with JC-1 (Abcam, ab141387) according to the manufacturer’s instructions. Mitochondrial JC-1 monomers (green) and aggregates (red) were detected by fluorescence microscopy and laser confocal microscopy.

### Measurement of ROS

L02 cells were inoculated into 6-well plates, transfected with siHADHA or siControl for 24 h, and then treated with 1 mM FFA for 24 h. The cells were incubated with 2ʹ,7ʹ-dichlorofluorescein diacetate in the dark for 0.5 h in the presence or absence of NAC (ROS scavenger N-acetylcysteine, 3 mM). The cells were washed to eliminate extracellular DCFH-DA with PBS, and intracellular DCFH-DA was transformed into fluorescent dichlorofluorescein (DCF). Then, DCF fluorescence of the cells was analysed by flow cytometry at 480 nm/520 nm.

### Biochemical analysis

The triglyceride (TG, NJBI, A110-1), alanine aminotransferase (ALT, NJBI, C009-2), aspartate transaminase (AST, NJBI, C101-2), adenosine triphosphate (ATP, NJBI, A095), hydrogen peroxide (H_2_O_2_, NJBI, A064-1), catalase (CAT, NJBI, A007-1-1) and total cholesterol (TC, NJBI, A111-1-1) levels in vivo and in vitro were measured according to the manufacturer’s instructions.

### HE staining

Tissue sections were stained with Mayer’s haematoxylin staining solution for 5–7 min, washed in ddH_2_O to turn blue, incubated with 1% hydrochloric acid alcohol for differentiation for 2–5 seconds, and washed with ddH_2_O. After air-drying, the slides were mounted with neutral gum. Finally, the morphologic changes in the liver tissues were observed under a light microscope.

### Statistical analysis

The data were analysed by SPSS 18.0 and are presented as the mean ± SEM (standard error of the mean). Significant differences between groups were determined by two-way ANOVA followed by Tukey post hoc tests. A P value < 0.05 was considered to indicate statistical significance.

## Results

### The expression of HADHA was decreased in FFA-treated cells and NAFLD mouse liver tissues

**Fig. 1:**
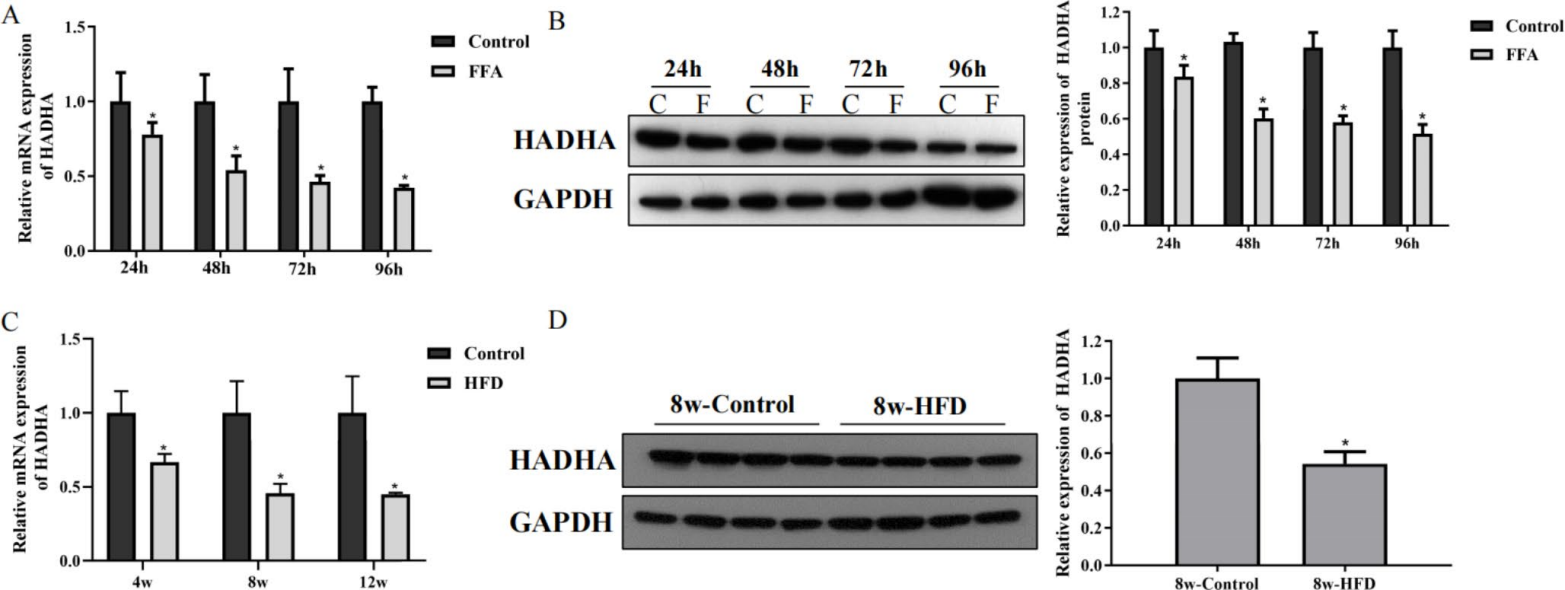
The expression of HADHA was decreased in FFA-treated cells and liver tissues of NAFLD mice. A-B: HADHA mRNA and protein levels in FFA-treated L02 cells were examined by qRT–PCR and western blotting. C-D: HADHA mRNA and protein levels in NAFLD mouse liver tissues were examined by qRT–PCR and western blotting. *P < 0.05 compared with the control group

To study the potential role of HADHA in the development of NAFLD, we measured the expression of HADHA in FFA-treated L02 and NAFLD mouse liver tissues. FFA treatments inhibited the expression of HADHA in L02 cells (Fig. 1 A and Fig. 1B). In addition, liver tissues from NAFLD patients showed decreases in HADHA mRNA and protein levels (Fig. 1 C and Fig. 1D). Together, these results demonstrated that HADHA may be related to the progression of NAFLD.

### Knockdown of HADHA accelerated hepatic steatosis in FFA-treated L02 cells

**Fig. 2:**
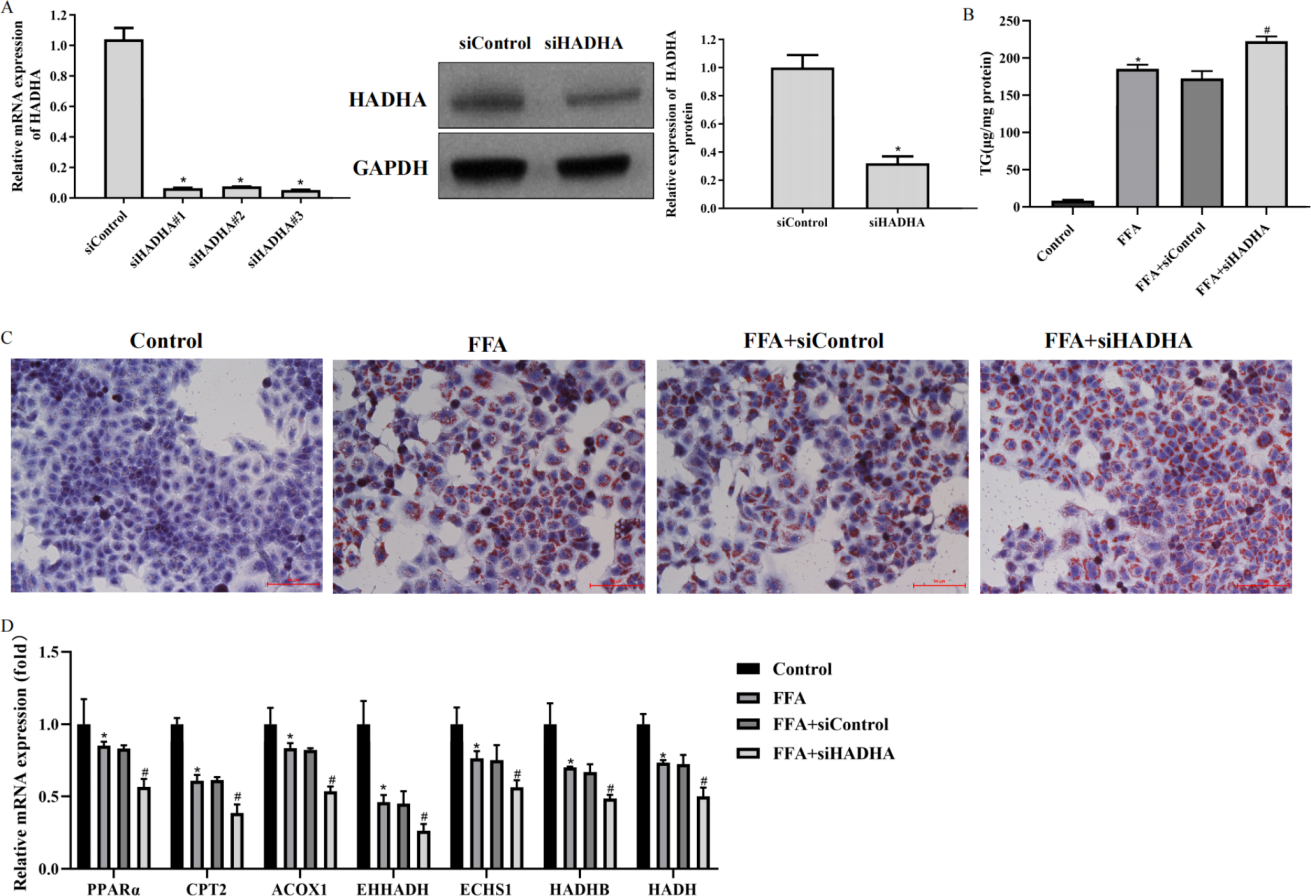
Knockdown of HADHA accelerated hepatic steatosis in FFA-treated L02 cells A: L02 cells were transfected with siHADHA or siControl for 24 h, and western blotting and qRT–PCR were used to analyse the protein and mRNA expression of HADHA. B: L02 cells were transfected with siHADHA or siControl for 24 h and then treated with 1 mM FFA for 24 h. TG kits were used to measure the TG content. C: Oil Red O staining assay detected the accumulation of lipid droplets in L02 cells (200×). D: qRT–PCR detected lipid metabolism-related gene expression. *P < 0.05 compared with the control group; ^#^P < 0.05 compared with the FFA + siControl group

To study the role of HADHA in the pathological process of NAFLD, we successfully constructed siHADHA, and L02 cells transfected with siHADHA had low expression of HADHA protein and mRNA (Fig. 2 A). High levels of TG were observed in FFA-treated L02 cells transfected with siHADHA (Fig. 2B). In addition, Oil red O staining showed increased lipid accumulation in FFA-treated L02 cells transfected with siHADHA (Fig. 2 C). Decreases in the expression of genes associated with lipid metabolism-related factors, including peroxisome proliferator-activated receptor-α (PPARα), carnitine palmitoyl transferase 2 (CPT2), acyl-CoA oxidase 1 (ACOX1), enoyl-CoA hydratase and 3-hydroxyacyl CoA dehydrogenase (EHHADH), enoyl-CoA hydratase, short chain 1 (ECHS1), hydroxyacyl-CoA dehydrogenase trifunctional multienzyme complex subunit beta (HADHB) and hydroxyacyl-CoA dehydrogenase (HADH), were detected in FFA-treated L02 cells transfected with siHADHA (Fig. 2D). Collectively, the findings above indicated that downregulation of HADHA accelerated lipid accumulation in NAFLD cells.

### Inhibition of HADHA accelerated mitochondrial dysfunction and oxidative stress in L02 cells

**Fig. 3:**
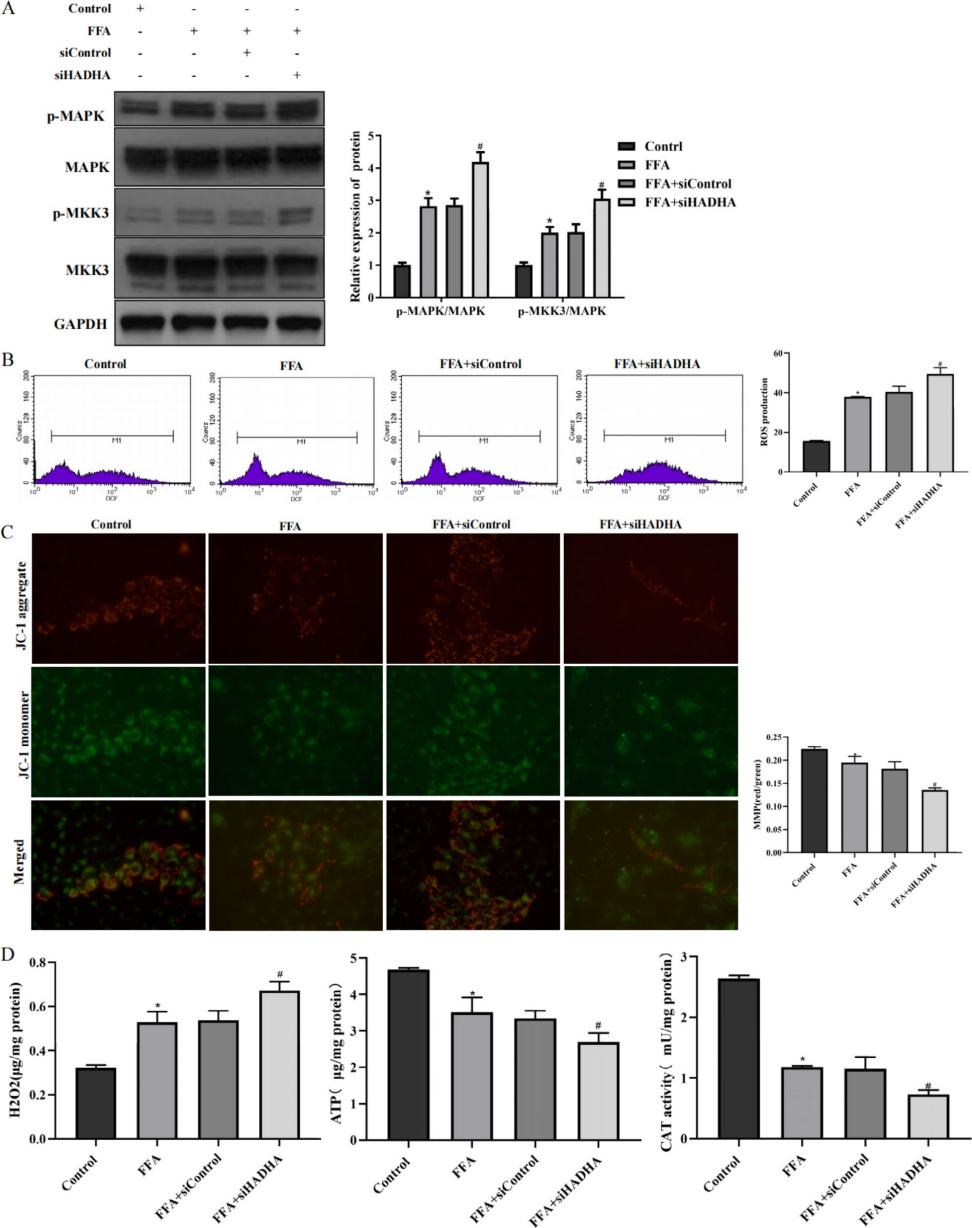
Inhibition of HADHA accelerated mitochondrial dysfunction and oxidative stress in L02 cells A: L02 cells were transfected with siHADHA or siControl for 24 h and then treated with 1 mM FFA for 24 h. The proteins p-MAPK, MAPK, MKK3 and p-MKK3 were examined by western blotting. B: DCF fluorescence staining was used to examine ROS production. C: A JC-1 assay was used to examine MMP (400×). D: The levels of H_2_O_2_, ATP, and CAT in L02 cells were examined by biochemical tests. *P < 0.05 compared with the control group; #P < 0.05 compared with the FFA + siControl group

Phosphorylation of MKK3 (p-MKK3) which is the key upstream signal of MAPK, increases in the NAFLD liver and inhibiting p-MKK3 alleviates hepatic lipid accumulation and exacerbate inflammation 31, 32. Therefore, the regulatory mechanism of HADHA on the activation of MKK3 and MAPK was explored. In L02 cells treated with FFA, the expression of p-MKK3 and p-MAPK was increased by HADHA inhibition (Fig. 3 A). MAPK regulates oxidative stress, which is related to NAFLD development 17, 33. Inhibition of HADHA resulted in high ROS production (Fig. 3B). Moreover, MMP decreased when HADHA was inhibited (Fig. 3 C). In addition, ATP content and CAT activity decreased when HADHA was downregulated, while H_2_O_2_ levels increased when HADHA was downregulated (Fig. 3D). In summary, the regulation of HDAHA in NAFLD was associated with MKK3/MAPK activation, mitochondrial function, and oxidative stress.

### Upregulation of HADHA alleviated hepatic steatosis and inflammation in NAFLD mice

**Fig. 4:**
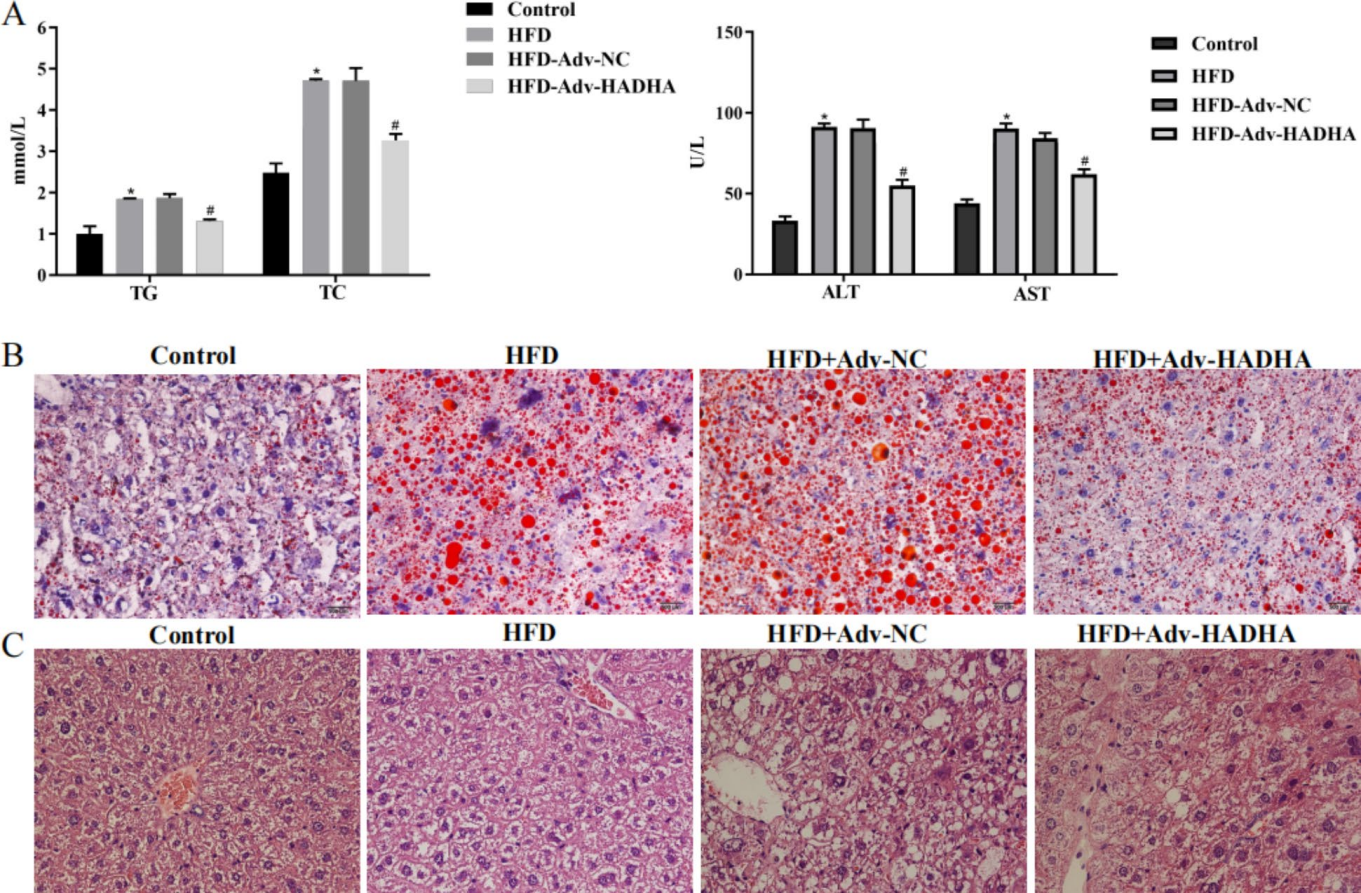
Upregulation of HADHA alleviated hepatic steatosis and inflammation in NAFLD mice. NAFLD mice were fed a HFD for 8 weeks, and Adv-NC or Adv-HADHA was used for tail vein injection once 2 weeks before sacrifice. A: TG, TC, ALT and AST in NAFLD mouse serum were examined by biochemical tests. B: An Oil Red O staining assay detected the accumulation of lipid droplets in mouse liver tissues (400×). C: An HE staining assay was used to analyse the liver tissue lesions of mice (400×). *P < 0.05 compared with the control group; #P < 0.05 compared with the HFD + Adv-NC group

To further verify the regulation of HADHA, NAFLD mice were fed a HFD for 8 weeks, and Adv-HADHA was used for tail vein injection. We found that the TG, TC, ALT and AST levels in NAFLD mouse serum were increased, while HADHA overexpression decreased the TG, TC, ALT and AST levels (Fig. 4 A). Oil Red O staining showed that NAFLD mouse liver tissues had large numbers of red-stained lipid droplets and that overexpression of HADHA decreased the numbers of red-stained lipid droplets in NAFLD mouse liver tissues (Fig. 4B). HE staining showed that the liver tissues of the NAFLD mice had increases in ballooning and degeneration of hepatocytes and visible focal necrosis, while overexpression of HADHA alleviated liver tissue lesions in NAFLD mice (Fig. 4 C). The above results indicate that upregulation of HADHA alleviated the abnormal lipid metabolism and inflammation of NAFLD mice.

### Upregulation of HADHA alleviated mitochondrial dysfunction and oxidative stress in NAFLD mice

**Fig. 5:**
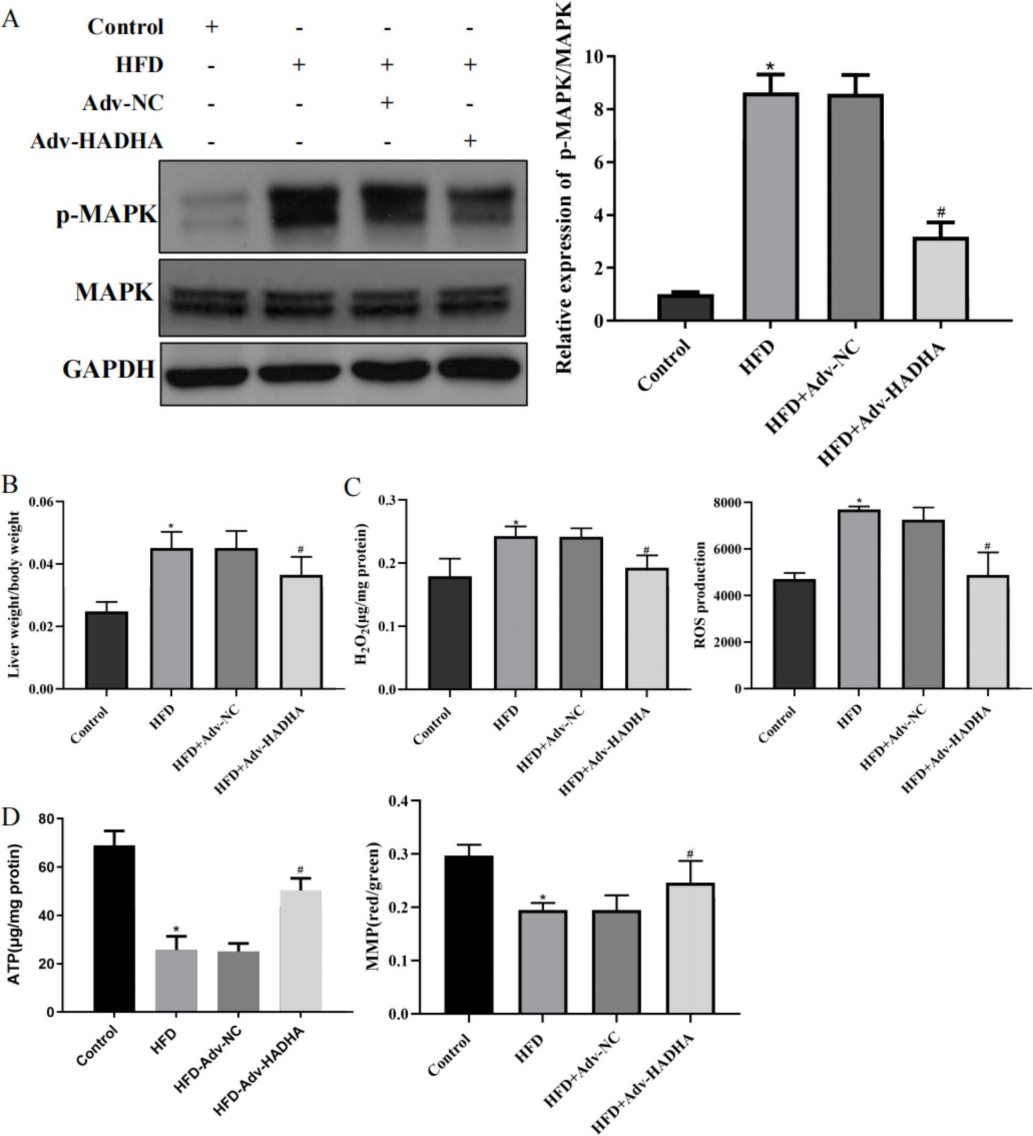
Upregulation of HADHA alleviated mitochondrial dysfunction and oxidative stress in NAFLD mice. NAFLD mice were fed a HFD for 8 weeks, and Adv-NC or Adv-HADHA was used for tail vein injection once 2 weeks before sacrifice. A: The expression of MAPK and p-MAPK in mouse liver tissues was examined by western blotting. B: The liver weight/body weight ratio was determined. C: H_2_O_2_ and ROS production were examined. D: ATP activity and MMP were examined. *P < 0.05 compared with the control group; ^#^P < 0.05 compared with the HFD + Adv-NC group

Moreover, Adv-HADHA effectively upregulated the expression of HADHA while downregulating p-MAPK in the liver tissue of NAFLD mice (Fig. 5 A). In addition, the liver weight/body weight ratio decreased when HADHA was upregulated (Fig. 5B). Moreover, the levels of H_2_O_2_ and ROS in the NAFLD mice were increased, while the ATP activity and MMP were reduced. The H_2_O_2_ and ROS levels in Adv-HADHA-treated NAFLD mice were reduced, while the ATP activity and MMP were increased (Fig. 5 C and Fig. 5D). In summary, HADHA improved the pathological changes in NAFLD liver tissue.

## Discussion

NAFLD, a metabolic disorder with a growing incidence, can progress to cirrhosis and liver cancer to threaten human life and health, but the mechanism of NAFLD is unclear 34. Apart from lifestyle changes and weight loss, there is a lack of an effective treatment strategy for NAFLD. Here, we identified HADHA as a key inhibitor of NAFLD. The expression of HADHA mRNA and protein was decreased in NAFLD mouse livers and human normal hepatocytes L02 stimulated by FFA. Knockdown of HADHA in L02 cells stimulated by FFA markedly promoted lipid accumulation, mitochondrial dysfunction, and oxidative stress. In contrast, overexpression of HADHA alleviated lipid accumulation, liver lesions, and oxidative stress in NAFLD mice. Finally, we showed that HFD feeding or FFA administration activated the MKK3/MAPK pathway, and this activation was reversed by downregulation of HADHA. In summary, our findings strongly suggest that targeting HADHA with the MKK3/MAPK pathway may attenuate NAFLD progression.

Growing evidence has shown that the pathogenesis of NAFLD is complicated and involves lipid accumulation, oxidative stress, mitochondrial dysfunction, and ER stress 35, 36, 37, 38. And the activation of MAPK is related to hepatic lipid deposition in patients with NASH and the pathogenesis of NASH-related fibrosis 37. For example, the activating the MAPK pathway exacerbates liver fibrosis by inducing inflammatory factor secretion and abnormal lipid metabolism 39. Downregulation of MAPK inhibits hepatic steatosis and inflammation in L02 cells 40. IL11 leads to hepatocyte death via NADPH oxidase 4 (NOX4)-derived ROS and activation of MAPK to impair mitochondrial function and inhibit fatty acid oxidation 41. Oleic acid upregulates the expression of hepassocin (HPS) to activate MAPK, leading to lipid accumulation in HepG2 cells 19. Consistent with our research, FFA-treated L02 cells had increased p-MAPK levels upon inhibition of HADHA, and these increases were accompanied by increases in ROS production, TG and H_2_O_2_. Moreover, our study showed that the expression of p-MAPK was decreased in HFD-challenged mouse livers when HADHA was overexpressed. MKK3, an upstream activator of p38 MAPK, increases p-MAPK expression, leading to the phosphorylation of MKK-3/6 and upregulation of ERK1/2 in macrophages 42, 43. In addition, the p-MKK3 protein was upregulated by HADHA inhibition in FFA-treated cells. Therefore, HADHA can inactivate MKK3/MAPK pathways to protect against NAFLD.

HADHA, a fatty acid β-oxidation-related factor, mediates lipid programming to regulate multiple cellular programs, such as apoptosis, fat metabolism and mitochondrial function 26, 44, 45, 46, 47. Increasing research has shown the important role of HADHA in metabolic dysfunction in the liver. Recent studies have demonstrated that downregulation of HADHA expression is related to oxidative stress, hepatic steatosis and mitochondrial function in the liver 28, 47. Therefore, we hypothesized that HADHA participates in NAFLD progression to maintain the homeostasis of lipid metabolism. CPT2 mediates the β-oxidation of long-chain acyl-CoA in the mitochondrial matrix 48. Abnormal lipid accumulation of hepatocytes increases ALT and AST content while decreasing ATP content 49,50. Consistent with our research, HADHA showed a positive correlation with the expression of CPT2. However, overexpression of HADHA decreased the content of H_2_O_2_, ALT and AST. In summary, HADHA had a protective effect on steatotic cells via inhibition of MKK3 activation and MAPK signalling pathways, subsequently alleviating lipid accumulation, inflammation, ROS production, and ER stress.

## Conclusion

In summary, our study revealed a protective effect of HADHA against NAFLD progression. HADHA inhibition significantly accelerated lipid accumulation, oxidative stress, mitochondrial dysfunction, and ER stress by activating MKK3, which subsequently activated the downstream MAPK signalling pathways to promote p-MAPK expression. Finally, overexpression of HADHA alleviated steatosis and liver damage. Therefore, inhibition of MKK3/MAPK pathways by HADHA may be an effective treatment for NAFLD. However, considering the complexity of NAFLD pathogenesis, more studies to determine new targets for NAFLD treatments are needed.

## Data Availability

The datasets used and/or analysed during the current study are available from the corresponding author on reasonable request.
